# Performance of criteria for selecting evolutionary models in phylogenetics: a comprehensive study based on simulated datasets

**DOI:** 10.1186/1471-2148-10-242

**Published:** 2010-08-09

**Authors:** Arong Luo, Huijie Qiao, Yanzhou Zhang, Weifeng Shi, Simon YW Ho, Weijun Xu, Aibing Zhang, Chaodong Zhu

**Affiliations:** 1 Institute of Zoology, Chinese Academy of Sciences, Beijing 100101, China; 2 Graduate University of Chinese Academy of Sciences, Beijing 100049, China; 3 UCD Conway Institute of Biomolecular and Biomedical Sciences, University College Dublin, Dublin 4, Ireland; 4 Centre for Macroevolution and Macroecology, Research School of Biology, Australian National University, Canberra ACT 0200, Australia; 5 School of Biological Sciences, University of Sydney, Sydney NSW 2006, Australia; 6 Zhongbei College, Nanjing Normal University, Nanjing 210046, China; 7 College of Life Sciences, Capital Normal University, Beijing 100048, China

## Abstract

**Background:**

Explicit evolutionary models are required in maximum-likelihood and Bayesian inference, the two methods that are overwhelmingly used in phylogenetic studies of DNA sequence data. Appropriate selection of nucleotide substitution models is important because the use of incorrect models can mislead phylogenetic inference. To better understand the performance of different model-selection criteria, we used 33,600 simulated data sets to analyse the accuracy, precision, dissimilarity, and biases of the hierarchical likelihood-ratio test, Akaike information criterion, Bayesian information criterion, and decision theory.

**Results:**

We demonstrate that the Bayesian information criterion and decision theory are the most appropriate model-selection criteria because of their high accuracy and precision. Our results also indicate that in some situations different models are selected by different criteria for the same dataset. Such dissimilarity was the highest between the hierarchical likelihood-ratio test and Akaike information criterion, and lowest between the Bayesian information criterion and decision theory. The hierarchical likelihood-ratio test performed poorly when the true model included a proportion of invariable sites, while the Bayesian information criterion and decision theory generally exhibited similar performance to each other.

**Conclusions:**

Our results indicate that the Bayesian information criterion and decision theory should be preferred for model selection. Together with model-adequacy tests, accurate model selection will serve to improve the reliability of phylogenetic inference and related analyses.

## Background

Among the rigorous methods of tree reconstruction that are available, maximum likelihood (ML) and Bayesian inference (BI) have dominated phylogenetic studies in recent years [1-6]. Both methods are based on the likelihood function, which needs explicit models of evolution to capture the underlying evolutionary processes in sequence data [1-6]. For DNA sequences, the models are the evolutionary characterisation of one nucleotide being replaced by another one. Although the models are simplifications of the "true" evolutionary processes and are clearly wrong [3,7,8], they are approximations that have been widely accepted. The assumed model of nucleotide substitution can exert a significant influence on phylogenetic estimation. This is an increasingly important concern in the modern genomic era, with the growing use of multiple loci that have probably been subject to different substitution processes [6].

A variety of nucleotide substitution models have been devised, most of which are special cases of the general time-reversible (GTR) model in which each of the six pairwise nucleotide changes can have a distinct rate, and the frequencies of the four nucleotides are allowed to take different values [9]. Common extensions to this model include parameters for a proportion of invariable sites (I) and for gamma-distributed rate heterogeneity among sites (Γ). In the last few years, many improvements have been explored, including models that account for differences among the three codon positions [10,11], pattern heterogeneity of the substitution process (e.g., [12]), among-site heterogeneity of rates (e.g., [13]), compositional heterogeneity among lineages (e.g., [14]), and site-specific rate variation through time (e.g., [15,16]).

Statistical methods are often used to identify the substitution model that best describes the given DNA sequence data. Model selection using software such as ModelTest [17], DT-ModSel [3], and jModelTest [18,19] has now become standard procedure in phylogenetic analysis [4,20]. Alternatively, model determination can be conducted using a reversible-jump Markov chain Monte Carlo approach in a Bayesian setting [9]. This differs from past practice when model choice was conducted without statistical justification or by choosing the most parameter-rich model available [6,8]. Model selection is considered important because the use of alternative models can change the results of phylogenetic analysis. It has effects not only on the estimation of model parameters (e.g., genetic distances and branch lengths; [2,21]), but also on estimates of bootstrap support and posterior probabilities [2]. Furthermore, misspecified models can lead to errors in phylogenetic inference, especially for trees with short internal branches [6,21-23].

One of the challenges facing researchers is how to select the most appropriate substitution model for a given dataset. There is now a range of procedures from which to choose, including the hierarchical likelihood-ratio test (hLRT) [24-26], Akaike information criterion (AIC) [27,28], Bayes factor [29-31], Bayesian information criterion (BIC) [32], posterior probability [29,33,34], decision theory (DT) [3], and the emerging approach of cross-validation [35,36]. All of these can be used to select the best-fit model from a set of candidates, but differ in specific algorithms which may ultimately give rise to differences in their performance (for further details, see Methods).

Of the four widely-used model-selection criteria in phylogenetics - the hLRT, AIC, BIC, and DT - the hLRT was once argued to be reasonably accurate and to perform better in general than the AIC and BIC, a conclusion drawn from analyses of simulated data comparing six models [8]. However, the hLRT has been demonstrated to have several disadvantages, such as a dependence on the starting point and the path through the hierarchy of models [37,38], which undermine and limit its performance in model selection. It has been established that both the BIC and DT tend to select simpler models than the AIC [3,4,8,39], while the hLRT particularly favours complex ones [3,40]. Applying them to empirical data, Pol [38] found that different best-fit models were selected by the hLRT and AIC for 16 out of 18 datasets. Ripplinger and Sullivan [4] found that the hLRT, AIC, BIC, and DT criteria often selected different models for the same real datasets, which was similar to the results of Abdo et al. [39] based on simulated data using parameters estimated from a rodent mtDNA dataset; in contrast, several empirical studies found that the BIC and DT often selected the same model [4,39]. However, there is a need for a comprehensive systematic study of the performance of model-selection criteria.

Here we present a study of the performance of the four model-selection criteria hLRT, AIC, BIC, and DT. Considering the biases in model selection revealed by previous studies (as described above) and the convenience of simulated data for theoretical investigation [41], we conducted a total of 14 simulations of 33,600 datasets. Our investigation was limited to the 24 fundamental substitution models from the GTR family, assuming a stationary, time-reversible, and homogeneous Markov process. Based on the best-fit models selected by these criteria for these simulated datasets, we examined for each criterion the success rate of recovering simulated models (its accuracy) and the number of different models selected across replicate datasets (its precision); the dissimilarity and model biases of these criteria (see Methods for details) were also examined and compared statistically. In addition, we examined dissimilarity in analyses of datasets that were simulated under a slightly more complex model based on a simple homogeneous codon-substitution process. On the whole, our study aims to provide a comprehensive evaluation of the performance of model-selection criteria.

## Results

### Accuracy

In the 14 simulations (Table 1; see Methods), the mean accuracy scores for the BIC and DT were higher than those for the hLRT and AIC. The ANOVA-LSD tests demonstrated that there were no significant differences for the pairs of hLRT-AIC and BIC-DT respectively; however, very significant differences existed for the other pairs such as hLRT-BIC (P < 0.01). The full results of the accuracy analysis are provided in Additional file 1.

**Table 1:** Conditions used in simulations for 24 models of the GTR family.

Simulation	Parameter set	Tree height	Ntaxa	Nchar	Simulation software	No.
I	A	0.7	30	1000	Seq-Gen	I-1
	A	0.5	30	1000	Seq-Gen	I-2
	A	0.3	30	1000	Seq-Gen	I-3
	A	0.1	30	1000	Seq-Gen	I-4
	B	0.7	30	1000	Seq-Gen	I-5
	B	0.5	30	1000	Seq-Gen	I-6
	B	0.3	30	1000	Seq-Gen	I-7
	B	0.1	30	1000	Seq-Gen	I-8
II	A	0.5	22	1000	Seq-Gen	II-1
	A	0.5	50	1000	Seq-Gen	II-2
III	A	0.5	22	1000	Mesquite	III-1
IV	A	Nonclock	22	1000	Seq-Gen	IV-1
V	A	0.5	30	300	Seq-Gen	V-1
	A	0.5	30	2000	Sen-Gen	V-2

One hundred replicates were performed for each set of conditions. The models consisted of JC (0) [61], K80 (1) [62], SYM (5) [63], F81 (3) [64], HKY (4) [65,66], GTR (8) [67], JC + I (1), K80 + I (2), SYM + I (6), F81 + I (4), HKY + I (4), GTR + I (9), JC + Γ (1), K80 + Γ (2), SYM + Γ (6), F81 + Γ (4), HKY + Γ (4), GTR + Γ (9), JC + I + Γ (2), K80 + I + Γ (3), SYM + I + Γ (7), F81 + I + Γ (5), HKY + I + Γ (5) and GTR + I + Γ (10), where 'I' represents the proportion of invariable sites, 'Γ' represents the discrete gamma distribution with four rate categories, and number in parentheses is the number of free parameters of each model. One classification of the 24 models was to put them into four categories: base (JC, K80, etc.), base + I (JC + I, K80 + I, etc.), base + Γ (JC + Γ, K80 + Γ, etc.) and base + I + Γ (JC + I + Γ, K80 + I + Γ, etc.). The other was that models with the same number of free parameters were grouped together, resulting in a total of 11 categories. In addition, we called every four models having the same parameters in the substitution-rate matrix as base-like models (e.g., the four models of SYM, SYM + I, SYM + Γ, SYM + I + Γ were called as SYM-like models).

**Figure 1:**
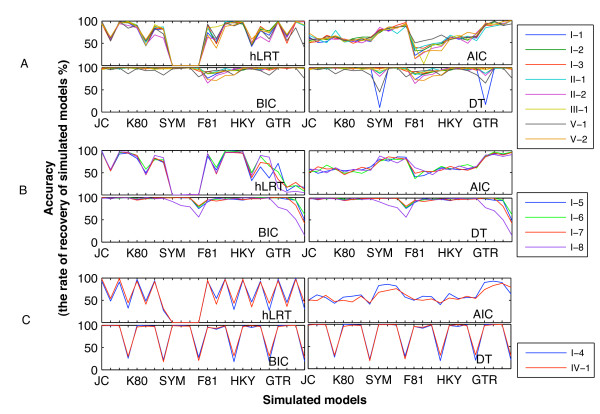
**Accuracy values of the four model-selection criteria for selecting 24 simulated models**. In the multiple-line charts, categories along the x-axis represent the simulated models. For the sake of clarity, only six models are labelled, and each one is followed by three similar ones (e.g., JC is followed by JC + I, JC + Γ, and JC + I + Γ). The y-axis represents the accuracy values (%). A shows the results of the simulations I-1, I-2, I-3, II-1, II-2, III-1, V-1 and V-2; B shows the results of the simulations I-5, I-6, I-7, and I-8; and C shows the results for the other two simulations.

**Figure 2:**
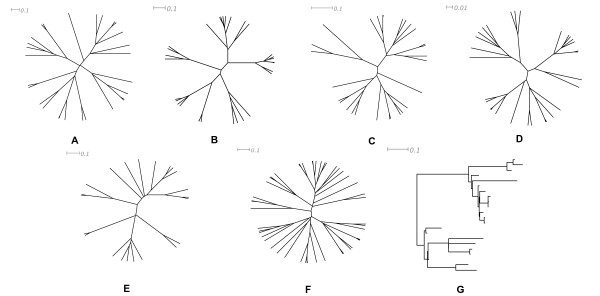
**Trees used to guide dataset simulations**. Tree heights are 0.7, 0.5, 0.3, and 0.1 substitutions per site for A, B, C, and D, respectively (30 taxa each); tree heights are 0.5 substitutions per site for both E (22 taxa) and F (50 taxa). All trees are ultrametric except for tree G, which is the non-clock tree (22 taxa) and was only used in simulation IV-1.

The hLRT exhibited high accuracy in recovering some models, but unexpectedly, it was always incapable of recovering the four SYM-like models (i.e., SYM, SYM + I, SYM + Γ and SYM + I + Γ; Table 1) (Figure 1). The AIC showed moderate or low accuracy except for a few complex models (e.g., GTR + I + Γ) for which the accuracy was even as high as 1.00 in certain simulations. The accuracy of the BIC and DT differed among simulations. In most cases, they showed high accuracy in recovering almost all of the 24 models (Figure 1A). Compared with the other models, however, two (SYM + I + Γ and GTR + I + Γ) were only moderately recovered in simulations derived from parameter set-B; even all of the SYM-like and GTR-like models were recovered less frequently in simulation I-8 (tree topology with a height of 0.1; Figure 2D) (Figure 1B). The BIC and DT exhibited similar accuracy in simulation I-4 (ultrametric tree topology of 30 taxa, 0.1 tree height; Figure 2D) and simulation IV-1 (non-clock tree topology of 22 taxa; Figure 2G), both recovering less than 35% of models of base + I + Γ category (i.e., JC + I + Γ, K80 + I + Γ, etc.; Table 1) (Figure 1C). In fact, they always selected models of base + Γ category (i.e., JC + Γ, K80 + Γ, etc.). Their accuracy values were high when the value of the parameter for proportion of invariable sites (*p*_*inv*_) was altered from 0.25 in parameter set-A to 0.5 in parameter set-B when simulating datasets (data not shown). Notably, the hLRT and AIC in these two simulations, especially the former criterion, also showed lower accuracy in recovering models of base + I + Γ category than in the other simulations.

### Precision

There were very significant differences among the precisions of the four criteria in the 14 simulations (randomized block ANOVA; P < 0.01). The precision of the hLRT was very significantly different from that of BIC and DT in certain simulations (P < 0.01), but not in others, while the precision of AIC was very significantly different from that of the other three criteria (LSD, P < 0.01) in all 14 simulations. The precision of BIC was always similar to that of DT, with P-values ranging from 0.508 to 1.000 (LSD). The full results of the analyses of precision are provided in Additional file 2.

**Figure 3:**
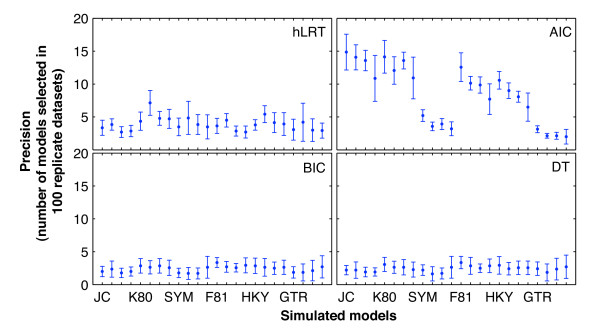
**Precision of the four criteria corresponding to 24 simulated models**. Categories along the x-axis represent the 24 simulated models. For the sake of clarity, only seven models are labelled, and each one is followed by three similar ones (e.g., JC is followed by JC + I, JC + Γ, and JC + I + Γ). The y-axis represents the means and standard deviations of precision values for each simulated model across the 14 simulations, which are different statistical results from those in Additional file 2. The markers denote the means, while lengths of error bars denote the standard deviation values.

Although small discrepancies existed, precision values of the AIC were generally higher than those of the other three in the 14 simulations (Figure 3). Their means ranged from 7.79 to 9.75, while standard deviations were also much larger and ranged from 4.169 to 5.160 (Additional file 2). This was mainly attributed to the fact that the AIC usually selected a dozen different best-fit models for each set of 100 replicates simulated under the same conditions, but at the same time, it selected only a few for datasets simulated under SYM-like and GTR-like models. Compared with the AIC, the other three criteria selected fewer different best-fit models, and their precision values were relatively stable among datasets generated under the same simulation conditions. However, precision values of the hLRT (means ranging from 3.29 to 4.83; Additional file 2) were generally higher than those of the BIC and DT, and in some cases were very significantly different. Therefore, the BIC and DT exhibited the best precision among the four criteria - lower mean and smaller standard deviation - while that of the BIC was little better than that of DT (Additional file 2).

### Dissimilarity

The percentages of one same model, two models, three models and four models being selected within each set of 100 replicate datasets were summarised, and Table 2 shows their means in each of the 14 simulations (Table 2). It was clear that two different best-fit models were generally estimated the most frequently by these criteria, with means ranging from 46.13% to 54.75%, followed by one same model from 33.83% to 47.67%, and three models from 5.17% to 12.88%. Four different models were favoured less than 0.25% by these four criteria in the 14 simulations, and even zero in I-4, IV-1, and V-2.

**Table 2:** Number of model(s) selected by the four model-selection criteria in the 14 simulations.

Simulation **No**.	One model (%)	Two models (%)	Three models (%)	Four models (%)
I-1	41.33	48.08	10.5	0.08
I-2	46.13	47.96	5.88	0.04
I-3	46.13	47.54	6.25	0.08
I-4	42.29	50.04	7.67	0
I-5	33.83	54.75	11.33	0.08
I-6	35.96	53.96	9.96	0.13
I-7	35.63	53.54	10.63	0.21
I-8	34.54	52.5	12.88	0.08
II-1	46.75	46.75	6.42	0.08
II-2	42.38	49.46	8.13	0.04
III-1	46.71	48.04	5.17	0.08
IV-1	37.83	52.67	9.5	0
V-1	43.75	47.38	8.75	0.13
V-2	47.67	46.13	6.21	0

Due to rounding, the four numerical values in some simulations do not sum exactly to 100%.

**Figure 4:**
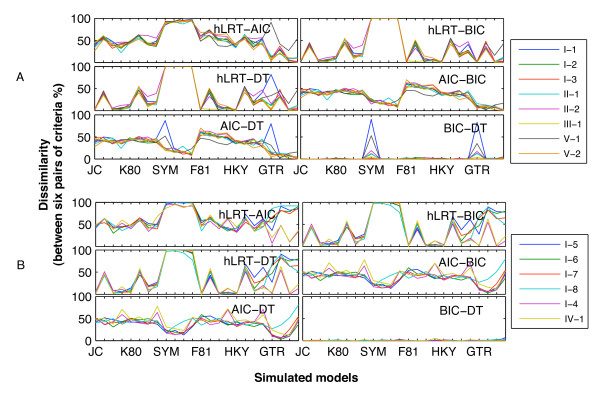
**Dissimilarity of six criterion pairs for 24 simulated models**. These charts illustrate the dissimilarity of every pair of model-selection criteria corresponding to 24 simulated models. Categories along the x-axis represent the 24 models. For the sake of clarity, only seven models are labelled, and each one is followed by three similar ones (e.g., JC is followed by JC + I, JC + Γ, and JC + I + Γ). The y-axis represents the dissimilarity values (%). A shows the results of the simulations I-1, I-2, I-3, II-1, II-2, III-1, V-1, V-2, while B shows the results of the other simulations.

Figure 4 illustrates the dissimilarity values of the six criterion pairs, and Additional file 3 shows the results of the ANOVA-LSD tests. In the 14 simulations, there existed very significantly different variances for different criterion pairs (P < 0.01), and also very significantly different variances for datasets simulated using the 24 different models. Dissimilarity values of the BIC-DT pair, with means ranging from 0.17% to 7.67%, were very significantly different from those of the other criterion pairs (P < 0.01). Although the dissimilarity of hLRT-AIC was less than that of certain pairs (e.g., hLRT-BIC) in datasets simulated for some models (e.g., SYM-like models; Figure 4), the means were the largest in the 14 simulations (ranging from 48.21% to 63.29%). Its values were very significantly different from those of the other pairs in seven simulations. Across the other simulations, their differences from some of the other criterion pairs were not very significant in spite of low probabilities. With simulation I-4 similar to IV-1, dissimilarity values in simulations derived from parameter set-B were similar to each other (Figure 4B); the other simulations derived from set-A generally resembled each other (Figure 4A).

In the additional simulation VI, which was performed using complex codon models, means of percentages that one same model, two models, three models, and four models were selected were 8.25%, 53.83%, 36.29%, and 1.63%, respectively. Among the criterion pairs, means of dissimilarity values ranged from 3.33% for BIC-DT to 84.88% for hLRT-AIC, and dissimilarity of BIC-DT was significantly different from that of the other criterion pairs (P < 0.01).

### Model biases

**Figure 5:**
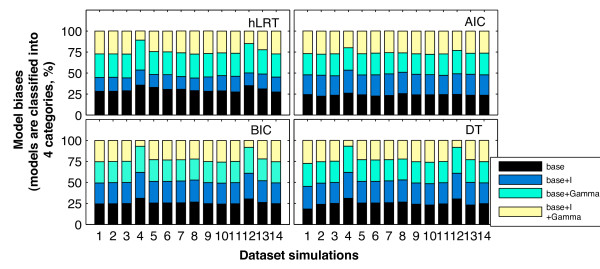
**General percentages of four model categories recovered**. The four stack-bar charts illustrate the percentages of base, base + I, base + Γ, and base + I + Γ in all recovered models of each simulation by every criterion considered. For the sake of clarity, numbers are labelled in the x-axis, representing the simulations in the order of I-1, I-2, I-3, I-4, I-5, I-6, I-7, I-8, II-1, II-2, III-1, IV-1, V-1, and V-2 from left to right.

While considering the four model categories (base, base + I, base + Γ and base + I + Γ; Table 1), the results of the chi-square (χ^2^) homogeneity tests [42] demonstrated that there were significant differences in model biases among the four criteria (α = 0.05) (Table 3). The hLRT was always significantly different from the other three (α' = 0.0083 by Bonferroni correction), with a relatively small portion of base + I category recovered in the 14 simulations (Figure 5). In contrast, the BIC was always similar to DT with high probabilities except in simulation I-1, where DT recovered fewer models of base category. For comparisons between the AIC and BIC, and between the AIC and DT, differences were generally not significant in simulations other than I-4, IV-1, and I-1. In fact, there was an even or an approximately even distribution of model categories recovered by the AIC, BIC, and DT in these simulations; but for I-4 and IV-1, models of base + I + Γ category were recovered much less by the BIC and DT than the other model categories (Figure 5).

**Table 3:** Statistics of χ^2 ^test and multiple comparison tests for the 14 simulations.

Simulation No.	**χ**^**2 **^**Sig.** (α = 0.05)	Multiple comparison Sig. (α' = 0.0083)					
		hLRT-AIC	hLRT-BIC	hLRT-DT	AIC-BIC	AIC-DT	BIC-DT
I-1	< 0.001	< 0.001	< 0.001	< 0.001	0.669	< 0.001	< 0.001
I-2	< 0.001	< 0.001	< 0.001	< 0.001	0.244	0.474	0.941
I-3	< 0.001	< 0.001	< 0.001	< 0.001	0.243	0.231	1.000
I-4	< 0.001	< 0.001	< 0.001	< 0.001	< 0.001	< 0.001	1.000
I-5	< 0.001	< 0.001	< 0.001	< 0.001	0.025	0.032	1.000
I-6	< 0.001	< 0.001	< 0.001	< 0.001	0.067	0.073	1.000
I-7	< 0.001	< 0.001	< 0.001	< 0.001	0.126	0.121	1.000
I-8	< 0.001	< 0.001	< 0.001	< 0.001	0.059	0.057	1.000
II-1	< 0.001	< 0.001	< 0.001	< 0.001	0.564	0.657	0.942
II-2	< 0.001	< 0.001	< 0.001	< 0.001	0.581	0.473	0.842
III-1	< 0.001	0.008	< 0.001	< 0.001	0.289	0.297	0.996
IV-1	< 0.001	< 0.001	< 0.001	< 0.001	< 0.001	< 0.001	1.000
V-1	< 0.001	< 0.001	< 0.001	< 0.001	0.012	0.058	0.128
V-2	< 0.001	< 0.001	< 0.001	< 0.001	0.740	0.763	1.000

The 24 models were classified into four categories: base, base + I, base + Γ, and base + I + Γ in these tests.

**Figure 6:**
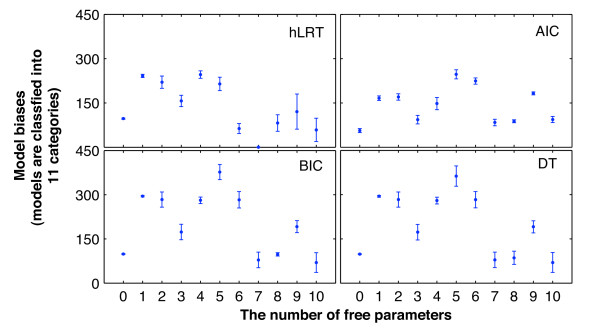
**Counts of models recovered, classified by the number of free parameters**. In these charts, the x-axis represents the numbers of model free parameters. The y-axis represents means and standard deviations of the counts for each of the 11 model categories across the 14 simulations. The markers denote the means, while lengths of error bars denote the standard deviation values.

Figure 6 shows the distribution of 11 model categories recovered in the 14 simulations based on the number of free parameters (Table 1). In all of them, with significant differences among the four criteria on the whole, each criterion was significantly different from any other except the pair of BIC and DT (see Additional file 4). However, there were also significant differences between the BIC and DT in simulation I-1.

## Discussion

### Which is the best criterion for model selection?

Although there exist cases indicating that obvious violation of model assumptions could favour the true tree [43,44] under specific conditions (e.g., oversimplified model for trees within the "Farris zone"; [45]), it has been clearly demonstrated that identifying the best-fit model is beneficial to phylogenetic inference and to understanding the molecular-evolutionary process. It must be acknowledged, however, that an absolute characterisation of the true evolutionary history of real data is usually impossible. So one concern for researchers, who do not have knowledge of this evolutionary history, is the accuracy of model-selection criteria to ensure that the best model can be selected from the available candidates.

In this study, based on 14 simulations of 33,600 datasets performed using known models, we simply evaluated the accuracy and precision of four model-selection criteria. Higher accuracy was broadly coincident with better precision and vice versa, which the performance of AIC best explained. Nevertheless, there were exceptions to this general pattern. Since the hLRT always selected GTR-like and TrN-like models for datasets simulated using SYM-like models, its precision values were low and its accuracy was almost zero. Generally, the high accuracy and low precision of the BIC and DT indicate that they perform better than the other two criteria. This result is robust to the influences of different simulation programs (Seq-Gen [46] and Mesquite [47]), tree topologies comprising different numbers of taxa (22, 30, and 50), and sequence lengths (300 bp, 1,000 bp, and 2,000 bp) (Table 1). However, accuracy values in certain simulations may confuse the situation to some extent, due to different simulation conditions as described below.

#### 1. Parameters

Between simulations derived from Parameter set-A and those derived from set-B, discrepancies in performance existed for both the BIC and DT (e.g., accuracy values for models of base + I + Γ category between I-4 and I-8) (Figure 1). Considering that altering *p*_*inv *_from 0.25 in simulation I-4 and IV-1 to 0.5 can improve the recovery of models of base + I + Γ category by the BIC and DT, *p*_*inv *_should be an important parameter influencing accuracy.

#### 2. Tree topology

The accuracy of the BIC and DT in simulations with a tree height of 0.1 did not support the general conclusions very well, being different from the simulations using tree heights of 0.3, 0.5, and 0.7 (Figure 1). At the same time, this happened in the simulation with the non-clock tree topology with both short and long branches. Given the lower accuracy of the other two criteria in these two simulations, we suspect that model selection might not be very effective for data of limited information content.

### Do they select different best-fit models?

Our study revealed that the means of dissimilarity values of the hLRT-AIC pair ranged from 48.21% to 63.29%, and were the largest across the 14 simulations even though they were not necessarily statistically larger than those of the other pairs. In contrast, the BIC and DT tended to select the same model, with significantly lower means of dissimilarity values ranging from 0.17% to 7.67% across the 14 simulations. Model selection for datasets simulated under complex codon models also yielded the largest dissimilarity for hLRT-AIC and the smallest for BIC-DT. Thus, we can envisage that researchers using the hLRT and AIC would frequently face a problematic situation in which these criteria would select different models. At the same time, as an extension of the BIC incorporating branch-length error [3], DT brings doubt as to whether estimating branch-length error, a measure of phylogenetic performance, would influence model selection.

On the whole, if one uses these four criteria to select models for given sequence data, variance in best-fit models could be encountered with the greatest possibility of two different models, followed by moderate possibilities of one same model, and three models; there is little or no chance of encountering four different best-fit models. These results are generally consistent with those of Ripplinger and Sullivan [4], whose results were based on 250 real sequence datasets for the criteria of hLRT, AIC, BIC, and DT. However, the results of our additional simulation VI supported a different order: two models, three models, one same model, and four models. In all respects, results of both empirical studies [4] and simulation studies (this study) suggest that model selection with these criteria will often produce inconsistent models, which could present a confusing situation for researchers.

### Are there model biases?

When considering the four model categories, one unexpected discovery was the relatively small portion of base + I category recovered in the 14 simulations, including those simulated with the *p*_*inv *_of 0.5 in parameter set-B. This result indicates that model selection with the hLRT is not sensitive to the proportion of invariable sites under the condition that there is no other among-site rate heterogeneity. In comparison, there was an approximately even distribution of the four model categories by the AIC, BIC, and DT in most simulations (Figure 5), which is consistent with the ideal even distribution given that the number of datasets simulated under models of any one category (i.e., 100 × 6 = 600) is the same as that of any other category. In a sense, we could relate the typical insensitivity of the hLRT to the proportion of variable sites with its special hierarchy of pairwise comparisons, because this parameter is the last-optimised parameter in the default hierarchy of ModelTest v.3.7 [17]. As it has been demonstrated that the hierarchy has an influence on the performance of hLRT [8,38], future work could investigate whether different hierarchies might lead to a different success rate in recovering models of base + I category.

Nevertheless, with our two standards of classifying the 24 GTR-family models, the results all confirmed that the BIC exhibited similar model biases to DT, with only a few exceptions. This was consistent with the results of accuracy, precision, and dissimilarity, which to some extent demonstrated that results based on our simulated datasets were reasonable and consistent.

## Conclusions

Overall, our performance analysis based on simulated datasets indicates that the BIC together with DT should be preferred for model selection in phylogenetics, although some of our results departed from this general finding owing to specific simulation settings such as values of the proportion of invariable sites. However, in view of the improvements on GTR models explored in recent years (see Background), it is possible that the results from most of our simulated data represent a poor reflection of real sequence data, which have almost certainly evolved under more complex conditions. Accordingly, we suggest here that model selection by the BIC or DT, together with model adequacy tests by parametric bootstrap [48,49] or posterior predictive distributions [1], might be the best approach. Further studies can be done with simulated datasets under more complex evolutionary models to understand the performance of these criteria and to enhance phylogenetic studies.

## Methods

### Dataset simulation

We used two different parameter sets to simulate datasets. For parameter set-A [2], which was mainly derived from a mitochondrial DNA analysis except for the transition/transversion ratio and the proportion of invariable sites [50], the settings were as follows (as appropriate for each model): base frequencies 0.35 A, 0.22 C, 0.18 G, 0.25 T; rates (relative to GT) 2.675 AC, 7.35 AG, 6.125 AT, 0.225 CG, 30.7 CT; transition/transversion (κ) 2.0; gamma shape parameter (α) 0.67256; and proportion of invariable sites (*p*_*inv*_) 0.25. Parameter set-B was chosen according to the settings of Posada and Crandall [8] (as appropriate for each model): base frequencies 0.35 A, 0.15 C, 0.25 G, 0.25 T; rates (relative to GT) 2 AC, 4 AG, 1.8 AT, 1.4 CG, 6 CT; transition/transversion (κ) 2.0; and gamma shape parameter (α) 0.5. We set the *p*_*inv *_as 0.5 in parameter set-B. Gamma-distributed rates in both parameter sets were modelled with four discrete categories in the simulations.

Initial simulations were conducted to explore the general performance of the model-selection criteria. First, we generated four ultrametric tree topologies of 30 taxa using the program PAML 4.1 [51] assuming a birth-death process (speciation rate 0.1, extinction rate 0.1, sampling fraction 1.0). Tree heights (i.e., expected number of substitutions per site from the root to each tip) were 0.7, 0.5, 0.3, and 0.1 for the four trees (Figure 2A, Figure 2B, Figure 2C, and Figure 2D, respectively). Then, for each combination of parameter set and tree topology, we used Seq-Gen 1.3.2 [46] to simulate 100 replicate datasets for 24 fundamental models of varying complexity from the GTR family (Table 1, simulation I). A sequence length of 1,000 bp was used because it was representative of empirical sequence lengths typically used in phylogenetic studies [2], and was sufficient to evaluate the performance of most model-selection criteria [8].

Other simulations were conducted to investigate the influence of certain simulation conditions on the performance of the model-selection criteria (Table 1). With other parameters fixed, the purpose of simulation II was to test the effect of varying the number of taxa. Simulation III employed the program Mesquite 2.6 [47] to simulate datasets to investigate the impact of different simulation programs. Simulation IV adopted one non-clock tree of 22 taxa (Figure 2G): following the method of Lemmon and Moriarty [2], the internal branches were randomly labelled from 0 to 18, and each branch was then assigned a branch length of 10^2*x*/18-3^, where *x *was the number assigned to that branch; similarly, the lengths of the external branches (randomly numbered from 0 to 21) were given by 10^2*x*/21-3^. In simulation V, sequence lengths were 300 bp and 2,000 bp, respectively, with the other simulation conditions consistent with simulation I-2.

### Model selection

The most widely-used program for model selection, ModelTest v.3.7 [17], along with DT-ModSel [3], were employed to select the best-fit model. Default settings were used in each program; some default settings may influence the performance of certain criteria, such as the hierarchy of pairwise comparison of models for the hLRT [8,38], but some may not, including the use of neighbour-joining (NJ) to generate a starting tree [8,39]. The 56 nested candidate models (i.e., a simpler model is one special case of a more general model), corresponding to the '7 schemes' in jModelTest [18,19], included the 24 models used in our simulations. After likelihood scores (*L*) under the 56 candidate models were computed by PAUP* v.4.0b10 [52] based on NJ trees, the hLRT together with the AIC and BIC was applied to model selection using ModelTest v.3.7 [17]; DT-ModSel was used for DT [3].

For the hLRT, the pairwise likelihood ratio test is given by

$$\delta =2(\ln L_{1}-\ln L_{0})$$

where *L*_0 _is the likelihood score under the null hypothesis (simple model) and *L*_1 _is the likelihood score under the alternative hypothesis (complex model). Although this is widely accepted for testing the fit of nested candidate models in a specific sequence, there are many possible ways to traverse the hierarchy of pairwise model comparisons [8,38,53]. We used the default hierarchy in ModelTest v.3.7 [17]. The LRT statistic approximately followed a standard χ^2 ^distribution. However, when the null fixed parameters were at the boundary of the parameter space of the alternative model (i.e., for tests of rate homogeneity among sites and invariable sites), the mixed χ^2 ^distribution (consisting of 50% $\chi_{0}^{2}$ and 50% $\chi_{1}^{2}$) was used to construct the tests [54-56]. We used 0.01 as the significance level for rejecting or failing to reject the null model.

The AIC is an asymptotically unbiased estimator of the Kullback-Leibler distance between the "true" model and the fitted model [27,57]. In contrast with the hLRT, the AIC can simultaneously compare all candidate models irrespective of their nesting status, and is defined as

$$\begin{array}{c} \text{AIC}=-2\ln L+2K\;\text{or}\\ {\text{AIC}}_{\text{c}}=-2\ln L+2Kn/(n-K-1). \end{array}$$

We computed the AIC instead of the AIC_c _for given models, in that the sample size *n *(i.e., the sequence length) for most of our simulated datasets was large enough compared with the number of parameters (*K*) [40,58]. The candidate model with the lowest AIC value was selected as the best-fit model.

Among the Bayesian methods of model selection, the BIC [32] is not limited to nested models, and allows the simultaneous comparison of multiple models [53]. It is computed as

BIC=−2lnL+Klnn,

where *n *is the sample size (i.e., the sequence length) and *K *is the number of parameters. Given equal prior probabilities of candidate models, the model yielding the smallest BIC was the one with the highest posterior probability and was selected as the best-fit one.

DT, a novel performance-based method of model selection, is an extension of the BIC and specifically incorporates branch-length error as a measure of phylogenetic performance in the course of model selection [3]. In DT-ModSel, DT estimated all candidate models through a penalty function, which was related to the difference in branch-length estimates across models. The model with the minimal posterior penalty was selected as the best-fit model.

### Performance analysis

#### Accuracy

For each set of 100 replicates, estimated best-fit models derived from each simulation were compared with the known model under which replicate datasets were simulated. We recorded the number of times that they matched (Number_matched_), and the accuracy of each criterion was then estimated by the rate of recovery of the simulated models, i.e.,

$$\text{Accuracy}={\text{Number}}_{\text{matched}}/{\text{Number}}_{\text{test}}\times 100\%,$$

where Number_test _was 100. The ANOVA for two-way randomised block design and LSD test were used to compare the accuracy of these four model-selection criteria.

#### Precision

We counted how many different best-fit models were selected by each criterion within each set of 100 replicates. We did not take account of how many times any particular model was selected among the 100 replicates, but simply interpreted the number of different models selected as an estimate of the precision of each model-selection criterion (i.e., we gave the same weight to each model selected). Therefore, the precision of these criteria for simulated models was approximately evaluated and was compared by the ANOVA for two-way randomized block design and LSD test.

#### Dissimilarity

To test whether the four criteria could select the same best-fit models for each replicate dataset or not, we counted the number of different models selected by the four criteria for each replicate dataset. We computed the percentages of one same model, two models, three models, and four models being chosen by the four model-selection criteria for each set of 100 replicates. In addition, we analysed the dissimilarity of two criteria based on the number of different models. For each pair of criteria, the number of instances of one same model (*m*) across each set of 100 replicates was counted, and their dissimilarity was computed as [59]

Dissimilarity=(N−m)/N×100%,

where *N *was equal to 100. The ANOVA for two-way randomized block design was used to compare the dissimilarity of the six pairs of criteria.

#### Model biases

Based on the success of recovery of the true model in each simulation (Number_matched_; see the '*Accuracy*' part), we employed the χ^2 ^homogeneity test [42] to compare the four criteria in terms of the composition of selected models. Here, we adopted two standards to classify the 24 simulated models (Table 1). Firstly, we classified them into four categories: base (JC, K80, etc.), base + I (JC + I, K80 + I, etc.), base + Γ (JC + Γ, K80 + Γ, etc.), and base + I + Γ (JC + I + Γ, K80 + I + Γ, etc.). These four model categories were then compared (with 3 degrees of freedom). Secondly, the 24 models were classified into 11 categories according to the number of free parameters (from 0 to 10; see Table 1 for details), and then analysed by the statistical test (with 10 degrees of freedom).

### Performance based on simulation under codon models

To evaluate the performance of the four criteria when the generating model is more complex than any of the 56 candidate models, we conducted another simulation (VI) as a special case using Recodon v.1.6.0 [60]. With the GY94 codon model [10] crossed with the settings in parameter set-A for the 24 fundamental models of GTR family respectively, and with the same base frequencies for all codon positions (1 × 4), 100 replicate datasets were simulated for each of the 24 simulated models here (i.e., a total 2,400). Other simulation parameters were set as sequence length 999 bp, number of sequences 30, a constant nonsynonymous/synonymous rate ratio (ω = 0.016) across sites, and mutation rate 0.001. Following model selection with the four criteria as above, the dissimilarity among the criteria was examined. Other aspects of performance were not evaluated, as those analyses were not practicable or were probably affected by the fact that the true model was not represented among the candidates.

## Authors' contributions

CZ, AL, AZ, and YZ conceived this study. AL, WS, AZ, and WX performed the work and the statistical analyses. HQ, WX, and SYWH participated in discussions and wrote code to facilitate computations. All authors read and approved of the final manuscript.

## Supplementary Material

Additional file 1 **Table of statistics of accuracy values in the 14 simulations**. The statistic variables include some derived from descriptive statistics, and the others from the ANOVA-LSD tests. The column 'LSD sig.' only shows criterion pairs that had no significant differences (α = 0.01). Click here for file

Additional file 2 **Table of statistics of precision values in the 14 simulations**. The column 'LSD sig.' only shows criterion pairs that had no significant differences (α = 0.01). Click here for file

Additional file 3 **Table of statistics of dissimilarity values in the 14 simulations**. For convenience of notation, in this table 'a' denotes the pair of hLRT and AIC; 'b', hLRT and BIC; 'c', hLRT and DT; 'd', AIC and BIC; 'e', AIC and DT; 'f', BIC and DT. It is noted that only pairs with significant differences are shown in the column 'LSD sig.' (α = 0.01). Click here for file

Additional file 4 **Table of statistics of χ^2 ^and multiple comparison tests for the 14 simulations**. The 24 models were classified into 11 categories according to the number of free parameters (from 0 to 10). Click here for file
